# Comparison of clinical safety and efficacy of ultrasound-guided local lauromacrogol injection versus uterine artery embolization in the treatment of caesarean scar pregnancy: a systematic review and meta-analysis

**DOI:** 10.1186/s12884-023-05455-2

**Published:** 2023-03-07

**Authors:** Ziwei Du, Wenjian Xu, Jingyuan Lu, Cheng Li

**Affiliations:** 1 Department of Interventional Radiology, Women’s Hospital of Nanjing Medical University, Nanjing Maternity and Child Health Care Hospital, Nanjing, 210009 China; 2 Department of Radiology, BenQ Medical Center, BenQ Hospital Affiliated of Nanjing Medical University, Nanjing, 210009 China

**Keywords:** Caesarean scar pregnancy, Ectopic pregnancy, Ultrasound-guided local lauromacrogol injection, Meta-analysis, Uterine artery embolization

## Abstract

**Background:**

The aim of this systematic review and meta-analysis was to introduce the relatively novel method of ultrasound-guided local lauromacrogol injection (USG-LLI) followed by dilatation and curettage for caesarean scar pregnancy (CSP) and to investigate the clinical safety and efficacy between uterine artery embolization (UAE) and USG-LLI in the treatment of CSP.

**Methods:**

The relevant literature and articles about USG-LLI, UAE and CSP published in eight electronic databases were searched to extract the primary outcomes for the selected articles. Review Manager Software(RevMan) V.5.2 was used for quantitative data synthesis and data analysis. Forest plots, sensitivity analysis and bias analysis were also performed on the included articles.

**Results:**

Of 10 studies included in our search, 623 patients were in the USG-LLI group and 627 patients were in the UAE groups. There were no significant differences between the two groups in terms of success rate, blood loss and time to human chorionic gonadotropin (hCG) normalization. However, USG-LLI group patients than UAE group patients had a shorter duration of hospital stay (mean difference [MD] = -1.97; 95% confidence intervals [*CI*] -2.63 to -1.31; *P* < 0.05; *I*^2^ = 95%), shorter restored menses (MD = -4.84; 95%*CI* -5.78 to -3.90; *P* < 0.05; *I*^2^ = 95%), and lower complication rates [odds ratio(OR) = 0.21; 95%*CI*:0.15 to 0.30; *P* < 0.05]; and cheaper on expenses of hospitalization (MD = -8028.29; 95%*CI* -10,311.18 to -5745.40; *P* < 0.05; *I*^2^ = 100%).

**Conclusions:**

The results demonstrate that USG-LLI is comparable in curative effect and success rates with UAE in the therapy of CSP, but patients in the USG-LLI group seem to have fewer complications rates, shorter duration of hospital stays and lower costs.

## Background

Caesarean scar pregnancy(CSP) is a relatively new and rare form of life-threatening of extrauterine pregnancy characterized by the implantation of a gestational sac into weak myometrial scar tissue in the lower anterior uterine wall of a previous caesarean section [1]. CSP patients were first reported by Larsen and Solomon in 1978 [2]. The actual incidence of CSP is unclear, however, recent studies have shown that the incidence of CSP ranges from 1/2216 to 1/1800, accounting for 6.1% of all extrauterine pregnancies or 0.15% of women with at least one caesarean delivery [3–5]. Although the World Health Organization (WHO) guidelines recommend that each country should have no more than 15% of caesarean births per year, however WHO figures shows that in 2007–2008, about half of all babies were born by caesarean section, and the percentage has increased in recent years, especially in developing countries [6, 7]. When early diagnosis is unclear and cannot be effectively treated, CSP can lead to massive bleeding, uterine rupture, hysterectomy, and even maternal death [8].

There are various treatment methods for CSP, which include expectant treatment, systemic or local methotrexate(MTX), dilatation and curettage (D&G), uterine artery embolization (UAE), hysterectomy or laparoscopy wedge excision; however, the optimal treatment regimen for CSP still remains individualized assessment and treatment [9, 10]. Several reports suggest that UAE combined with curettage has been recommended as a minimally invasive and effective treatment [11, 12]. Nevertheless, UAE is not only limited by specialized equipment and professionals, but also has many adverse reactions, such as Asherman's syndrome, uterine necrosis, radiation exposure, etc. [13, 14].

Lauromacrogol is a foamed clinical chemical sclerosants drug that has been widely used for varicose veins and cystic thyroid nodules [15, 16]. Lauromacrogol mainly uses its sclerosant effect to directly damage vascular endothelial cells, promote local thrombosis, adhere to blood vessels at the injection site, cause aseptic inflammatory lesions and tissue fibrosis, and replace the pathological blood vessels with fibrotic cords, resulting in sclerosis permanent occlusion of the purpose [17, 18]. The safety of lauromacrogol has been confirmed in gynecological surgery [19, 20]. Additionally, Chai et al. [21] first reported a new method of ultrasound-guided local lauromacrogol injection (USG-LLI) combined with curettage for CSP. Relevant studies have shown that the therapy is effective and safe [22, 23].

This meta-analysis aimed to investigate the clinical safety and efficacy of USG-LLI combined with curettage and UAE combined with curettage in the treatment of CSP. The meta-analysis presented the following article in accordance with the PRISMA reporting checklist [24]. Our research has been registered with PROSPERO, the code is CRD42022371579.

## Methods

### Literature search

Electronic databases searches for relevant articles were conducted in four English databases and four Chinese databases, including PubMed, Cochrane Library, Embase, Web of Science, Chinese BioMedical Literature Service System (SinoMed), Chinese Sci-Tech Journal Database(VIP), WanFang data and Chinese National Knowledge Infrastructure (CNKI). The articles were published online before 1 April 2022. The language of articles was limited to English and Chinese. For all index terms below, synonyms will be linked to a specific group by the Boolean operator "OR" instead of linking all groups by the Boolean operator "AND": ("artery embolization uterine" OR "embolization uterine artery" OR "uterine artery embolizations") AND ("Polidocanol" OR "lauromacrogol") AND ("Pregnancies" OR "Gestation" AND ("Cicatrix" OR ("Scar" OR "Scars" OR "Cicatrization" OR "Scarring")) AND ("cesarean sections" OR "abdominal deliveries" OR "deliveries abdominal" OR "caesarean section" OR "caesarean sections" OR "abdominal delivery" OR "C-Section" OR "postcesarean section" OR ("C" AND "Section") OR ("C-Sections")). This meta-analysis data is based on published articles.

### Study Selection

The articles retrieved in the subsequent analysis must meet the following criteria:

1. CSP patients received USG-LLI or UAE treatment
2. Case–control study, cohort case-series study, and randomized-controlled trial design
3. USG-LLI versus UAE for CSP treatment
4. Detailed reports available, such as mean and standard deviation or dichotomous data on efficacy and safety

Individual case reports or studies patients without clear data or patients receiving other treatments were excluded.

### Data extraction and quality assessment

Two independent reviewers (DZW and LJY) extracted the following data from the full-length studies retrieved: author's last name, country, year and month of publication, trial design, sample size, and patient demographics. We also extracted the following data for statistical analysis: gestational interval, gestational age, menstrual recovery time, gravidity, number of previous cesarean section, adverse reactions, blood loss, β-human chorionic gonadotropin (β-hCG) normalization time, treatment success rate, the length of hospital stay and treatment cost.

The quality of the included non-randomized controlled studies was assessed by using the Newcastle–Ottawa Scale(NOS), which ranges from zero to nine (assigned with an asterisk) and includes three aspects (patient selection, comparability of study groups, and assessment of outcome). Studies with a NOS score of 6 stars or more are considered to be of high quality. A third investigator (XWJ) addresses any discrepancies between the two investigators. To maintain the quality of the meta-analysis and reduce risk of bias, studies rated as low quality were removed.

### Statistical analysis

All statistical analyses and generation of forest plots were performed using RevMan V5.3 software (Cochrane Collaboration, London, UK). Results for continuous or dichotomous data were expressed as mean difference (MD) or odds ratio(OR) with 95% confidence intervals(CIs), respectively. The χ2 test was used to test for the heterogeneity of the studies based on the *Q* test and *Ι*^2^. According to Cochrane evaluation guidelines, when *Ι*^2^ > 50% or *Ρ* < *0.1,* we used a random-effects model (DerSimonian-Laird method) [25]; Otherwise, we used a fixed-effects model (Mantel–Haenszel method) [26]. Sensitivity analysis and publication bias were performed using the STATA version 12.0 (Stata Corp., College Station, TX, USA).

## Results

### Selection of literature

**Fig. 1:**
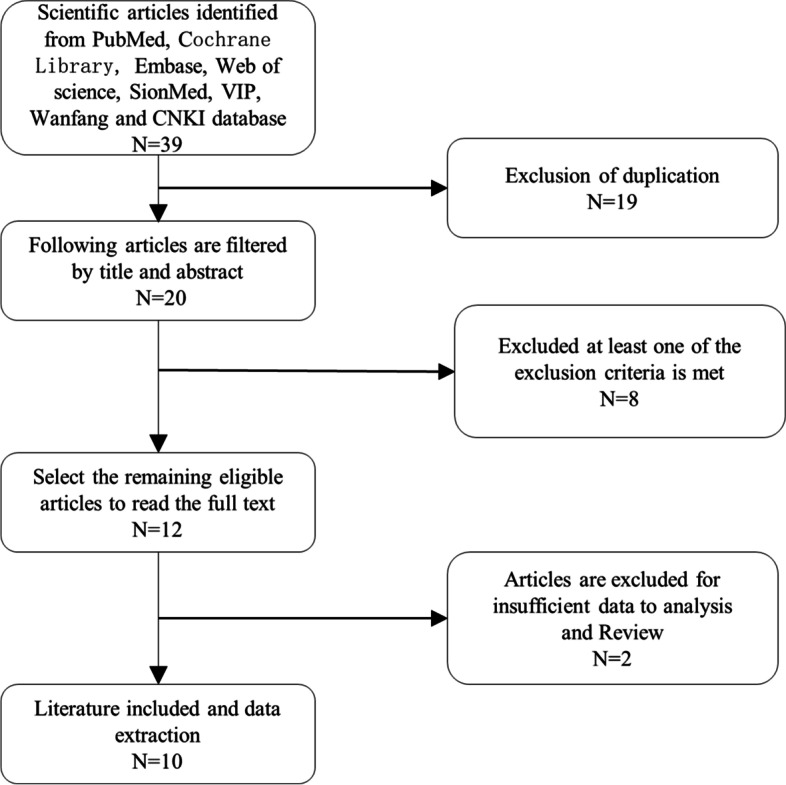
Flow diagram of the study selection process

Thirty-nine potentially relevant articles were found at a preliminary stage by searching the electronic databases. Filtering the titles and abstracts, we excluded 19 duplicate records and 8 records that met at least one of the exclusion criteria. After retaining twelve studies for the full-text review, one article with insufficient data and one review were excluded. Finally, ten records (4 English and 6 Chinese articles) that met all our inclusion criteria were included in our meta-analysis [22, 23, 27–34]. A flow diagram with relevant details of the study selection process is presented in Fig. 1.

### Study baseline characteristics and quality assessment

A total of 1250 patients with CSP were included in 10 studies, of which 623 were in USG-LLI group and 627 in UAE group. All articles refer to Chinese hospitals. As shown in Table 1, the key baseline characteristics of the ten studies are listed below.

**Table 1:** Baseline characteristics of ten studies included in the meta-analysis

Study	Year	Study design	Treatment regimen	NO.of patients	Age(mean ± SD, years)	Pregnancy duration(mean ± SD, days)	CS interval(mean ± SD, years)	Gravidity(n)	NO. of previous cesarean section
Dong et al	2018	Case–control	USG-LLI + curettage	78	35.87 ± 4.29	61.23 ± 12.3	NR	3.03 ± 0.78	NR
			UAE + curettage	68	36.12 ± 4.2	60.89 ± 12.63	NR	2.98 ± 0.82	NR
Hong et al	2022	Case–control	USG-LLI + suction aspiration	90	34.4 ± 5	46.2 ± 7.1	6 ± 3.4	NR	1.26 ± 0.75
			UAE + suction aspiration	160	33.1 ± 4.8	51.1 ± 12.3	5.7 ± 3.8	NR	1.14 ± 0.5
Qian et al	2020.02	Case–control	USG-LLI + curettage	61	33.47 ± 4.2	60.22 ± 13.78	NR	2.89 ± 0.92	1.33 ± 0.46
			UAE + curettage	70	32.56 ± 3.95	59.38 ± 14.32	NR	3.17 ± 0.59	1.12 ± 0.55
Qian et al	2020.07	Case–control	USG-LLI + curettage	30	33.56 ± 4.88	49.43 ± 12.07	NR	NR	1.37 ± 0.49
			UAE + curettage	30	33.06 ± 4.88	50.1 ± 10.13	NR	NR	1.37 ± 0.49
Wu et al	2020	Case–control	USG-LLI + curettage	86	33.17 ± 3.53	48.51 ± 12.84	5.82 ± 2.97	NR	1.57 ± 0.6
			UAE + curettage	65	34 ± 3.81	50.48 ± 9.12	4.96 ± 3.3	NR	1.63 ± 0.49
Xu et al	2020	RCT	USG-LLI + vacuum curettage	85	33.17 ± 3.53	60.27 ± 11.06	4.36 ± 1.84	2.76 ± 0.71	NR
			UAE + vacuum curettage	85	36.87 ± 1.08	58.59 ± 11.83	4.52 ± 1.92	2.82 ± 0.74	NR
Zhang et al	2020	Case–control	USG-LLI + curettage	86	32.1 ± 5.7	48.3 ± 12.1	NR	3.1 ± 1.8	NR
			UAE + curettage	39	32.9 ± 4.6	48.7 ± 10	NR	3.3 ± 1.5	NR
Zhang et al	2021	Case–control	USG-LLI + curettage	17	34.76 ± 5.99	61.88 ± 30.5	5.16 ± 2.5	NR	NR
			UAE + curettage	17	32.17 ± 2.29	52 ± 34.28	4 ± 2.5	NR	NR
Zhu et al	2021	RCT	USG-LLI + curettage	18	3.86 ± 18	47.28 ± 15.7	6.56 ± 3.37	3.5 ± 3	1.32 ± 0.25
			UAE + curettage	21	32.86 ± 4.91	52.57 ± 9.21	5.76 ± 3.78	3 ± 1.5	1.44 ± 0.5
Zhu et al	2022	Case–control	USG-LLI + curettage	72	32.28 ± 4.88	45 ± 8.15	4.5 ± 4.2	4 ± 0.5	1.3 ± 0.5
			UAE + curettage	72	33.04 ± 4.22	46 ± 8.89	6 ± 4.44	4 ± 0.5	1.43 ± 0.5

The quality assessment of the articles was conducted using the NOS. The assessment revealed that two of the studies had scores of 6 and 7(moderate risk of bias), whereas the remaining studies had a score of 8(low risk of bias) (Table2).

**Table 2:** Quality assessment of included 10 studies

Study	Selection	Comparability	Outcomes	Total
Dong2018.01	2	1	3	6
Hong2022.01	3	2	3	8
Qian2020.02	3	2	3	8
Qian2020.07	2	1	3	6
Wu 2020.11	3	2	3	8
Xu 2020.04	3	2	3	8
Zhang2020.03	3	1	3	7
Zhang2021.12	3	2	3	8
Zhu 2021.01	3	2	3	8
Zhu 2022.01	3	2	3	8

### Time of Serum β-hCG Normalization

**Fig. 2:**
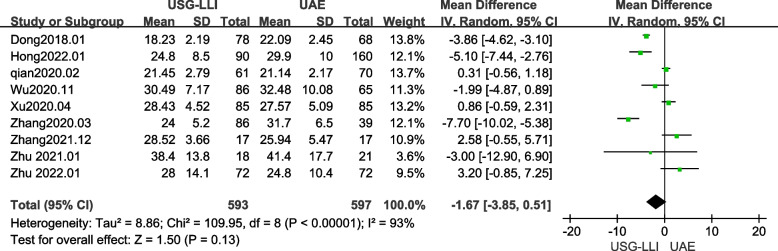
Forest plot showing the comparison normalization time of serum β- hCG between two groups

Nine articles reported the time to normalization of serum β-hCG [22, 23, 27–32, 34]. As shown in Fig. 2 (*P* < 0.00001, *I*^2^ = 93%), there was significant heterogeneity among these findings in our meta-analysis. Therefore, we employed a random-effects model to estimate the difference between the UAE and USG-LLI groups. Furthermore, the pooled data showed that the normalization time of serum β- hCG was approximately similar between two groups (MD = -1.67; 95%*CI*-3.85 to 0.51; *P* = 0.13).

### Blood loss

**Fig. 3:**
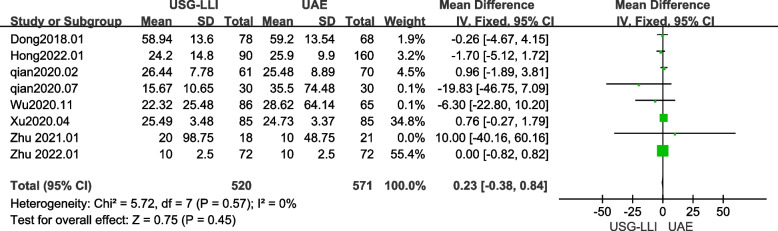
Forest plot showing the comparison blood loss during dilatation and curettage after UAE or USG-LLI groups

Eight out of ten papers reported the amount of blood loss during dilatation and curettage after UAE or USG-LLI [22, 23, 27–29, 32–34]. As shown in Fig. 3 (*P* = 0.57, *I*^2^ = 0%), there was no significant heterogeneity among these findings in our meta-analysis. Therefore, we employed a fix-effects model to estimate the difference between the UAE and USG-LLI groups. In addition, Fig. 3 and Table 3 show that the blood loss in patients receiving USG-LLI was approximately similar to that in patients receiving UAE (MD = 0.23; 95%*CI*-0.38 to 0.84; *P* = 0.45).

**Table 3:** Summary of results

Outcomes	Case/control	*P* for heterogeneity	Model	MD/OR	95%*CI*	*P*
Time to HCG normalization(d)	593/597	< 0.0001	Random	-1.67	-3.85,0.51	0.13
Blood loss(ml)	520/571	0.57	Fix	0.23	-0.38,0.84	0.45
Duration of the hospital stay(d)	623/627	< 0.0001	Random	-1.97	-2.63,-1.30	< 0.00001
Complication rate(%)	63/400; 121/325	0.31	Fix	0.22	0.15,0.33	< 0.00001
Success rate(%)	603/623; 600/627	0.96	Fix	1.41	0.79,2.51	0.25
Total cost(yuan)	533/467	< 0.0001	Random	-8308.62	-11,176.37, -5440.88	< 0.00001
Restored menses(d)	189/145	0.05	Random	-4.87	-5.82,-3.91	< 0.0001

Case: USG-LLI, Control: UAE,

Enumeration data: using events/total and odds ratio(OR); Yuan: Chinese currency unit

### Duration of hospital stay

**Fig. 4:**
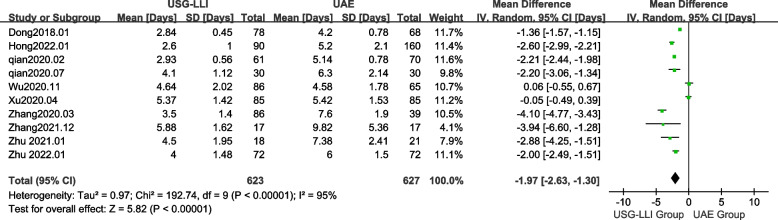
Forest plot showing the comparison duration of hospital stay between two groups

All articles reported the duration of hospital stay [22, 23, 27–34]. As shown in Fig. 4 (*P* < 0.0001, *I*^2^ = 95%), there was significant heterogeneity among these findings in our meta-analysis. Therefore, we employed a random-effects model to estimate difference between the UAE and USG-LLI groups. In Fig. 4 and Table3, patients receiving USG-LLI had significantly shorter hospital stays (MD = -1.97; 95%*CI*-2.63 to -1.30; *P* < 0.00001) than patients receiving UAE.

### Complication rate

**Fig. 5:**
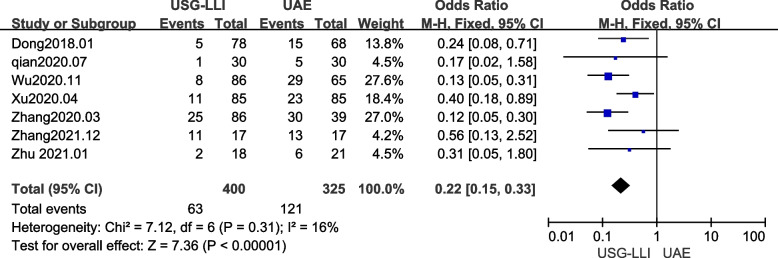
Forest plot showing the comparison complication rates between two groups

Complication rates were reported in seven out of ten papers [22, 28, 30–34]. Figure 5 (*P* = 0.31, *I*^2^ = 16%) shows that there was no significant heterogeneity among these findings in our meta-analysis. Therefore, we employed a fix-effects model to estimate the difference between the UAE and USG-LLI groups. As shown in Fig. 5 and Table 3, patients treated with USG-LLI had a significantly lower complication rate (*OR* = 0.22; 95%*CI* 0.15 to 0.33; *P* < 0.00001) compared with patients treated with UAE.

### Success rate

**Fig. 6:**
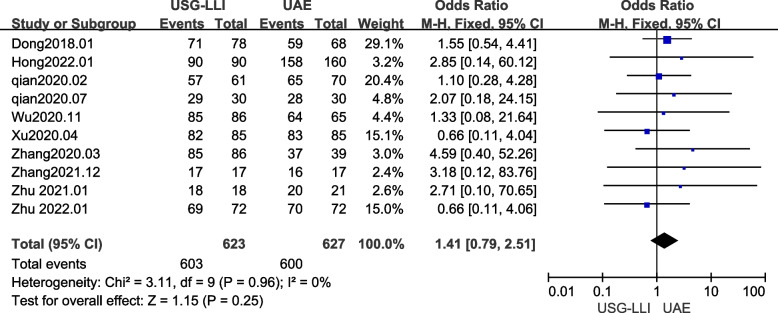
Forest plot showing the comparison success rates between two groups

The success rate of treatment for CSP using USG-LLI or UAE was reported in all papers [22, 23, 27–34]. As shown in Fig. 6 (*P* = 0.96, *I*^2^ = 0%) there was no significant heterogeneity among these findings in our meta-analysis. Therefore, we employed a fix-effects model to estimate the difference between the UAE and USG-LLI groups. As shown in Fig. 6 and Table 3, the success rate of patients who underwent USG-LLI was approximately similar to that of patients who underwent UAE (*OR* = 1.41; 95%*CI* 0.79 to 2.51; *P* = 0.25).

### Expenses of hospitalization

**Fig. 7:**
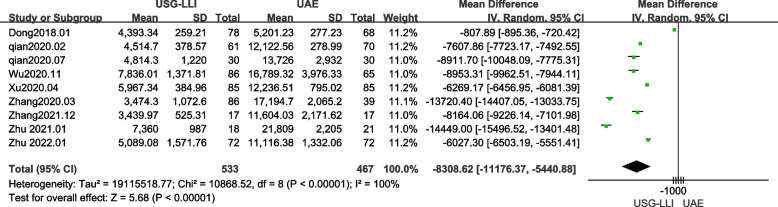
Forest plot showing the comparison expenses of hospitalization between two groups

Nine articles reported hospitalization expenses [22, 23, 28–34]. As shown in Fig. 7. (*P* < 0.0001, *I*^2^ = 100%), there was significant heterogeneity among results of our meta-analysis. Therefore, we employed a random-effects model to estimate the difference between the UAE and USG-LLI groups. In Fig. 7 and Table 3, hospitalization costs of the USG-LLI group were lower (MD = -8308.62; 95%*CI* -11,176.37 to -5440.88; *P* < 0.0001) than those of the UAE group.

### Restored menses

**Fig. 8:**
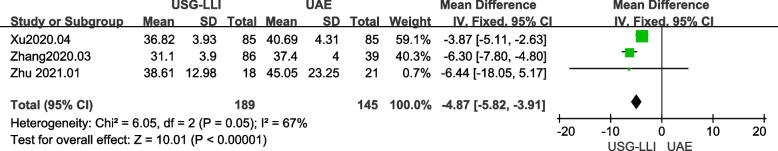
Forest plot showing the comparison the day of restored menses between two groups

Three out of ten articles reported days to menstrual recovery [28, 31, 34]. As shown in Fig. 8 and Table 3 (*P* = 0.05, *I*^2^ = 67%), there was significant heterogeneity among these findings in our meta-analysis. Figure 8 and Table 3 show that the menstrual recovery days of USG-LLI were significantly shorter than those of UAE group (MD = -4.87; 95%*CI* -5.82 to -3.91; *P* < 0.0001).

### Sensitivity analysis and publication bias

We assessed the robustness and strength of the pooled results by a sensitivity analysis excluding low-quality trials, which increased the confidence in the results of our meta-analysis.

**Fig. 9:**
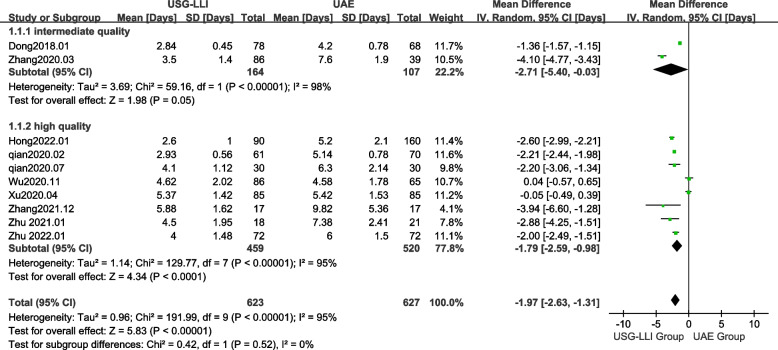
Forest plot showing the subgroup analysis of literature quality

To explore whether literature quality was a source of heterogeneity, we also performed subgroup analyses according to literature quality. We classified the included literature into high-quality group (MD = -1.79, 95%*CI* -2.59 to -0.98, *P* < 0.0001; *I*^2^ = 95%) and moderate quality group (MD = -2.71, 95%*CI* -5.40 to -0.03, *P* = 0.05; *I*^2^ = 98%) according to the criteria of NOS scoring system. As shown in Fig. 9, the test for difference between groups (high versus intermediate quality studies) was not statistically significant (*P* = 0.52).

**Fig. 10:**
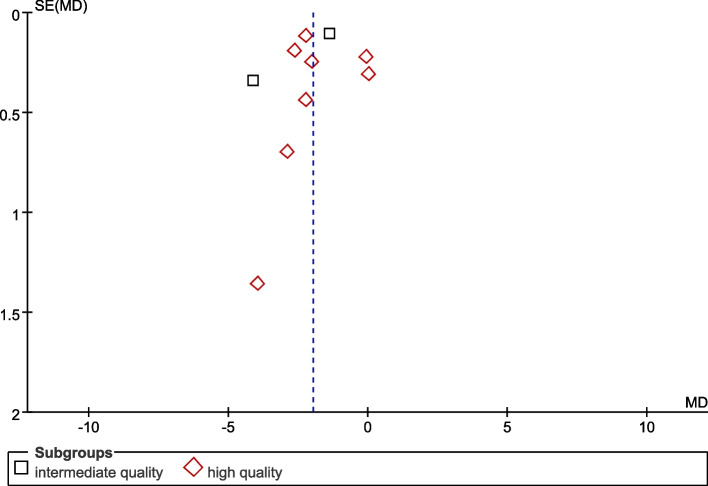
Publication bias funnel plot

Publication bias was explored using funnel plots generated by RevMan software. The funnel plot is symmetric and there is no clear evidence of publication bias (Fig. 10).

## Discussion

To our knowledge, this systematic review and meta-analysis is the first to assess the clinical safety and efficacy of USG-LLI and UAE followed by curettage in the treatment for CSP. Recently a growing number of recent studies [22, 23] have reported that USG-LLI is a safe, cost-effective treatment for CSP. Our meta-analysis showed that both USG-LLI and UAE had an impact on CSP treatment. However, the USG-LLI group had lower complications rates, shorter hospital stays, shorter intervals between menstrual recovery, and lower hospitalization costs. Therefore, our meta-analysis indicated that USG-LLI seems to be more suitable as an adjuvant therapy for CSP patients.

CSP is one of the potentially life-threatening long-term complications after a caesarean section, which is a gestational sac implanted in scarred area of the uterus. Because the gestational sac grows in weak scar muscle wall tissue, it may trigger serious complications, such as massive bleeding, uterine rupture, and even the death of the pregnant women [35]. There are multiple treatments for CSP; however, there is still no consensus on the treatment guidelines for CSP management. Moreover, the treatment experience was based more on recommendations from case reports based on the classification of CSP, severity of symptoms, hCG levels, and relevant experience [36, 37]. With the improvement of transvaginal contrast-enhanced ultrasonography and the continuous deepening of the understanding of the CSP, the experience of early diagnosis of CSP and effective treatment of CSP is gradually accumulating and progressing [38].

This meta-analysis suggested that the time to return to menstruation was significantly shorter in the USG-LLI group than in the UAE group. This may be due to the fact that local lauromacrogol injection only causes regional vessel occlusion and compression around the lesion, which protects ovarian function from damage, enables rapid recovery of the menstrual cycle, and preserves reproductive function [12, 21].

The incidence of complication rates in the UAE group was significant higher than in the USG-LLI group, which may be related to the fact that UAE procedure is an invasive treatment that may cause embolic syndrome, that is, the occurrence of lower abdominal pain, fever, and even infection. Thus, USG-LLI is associated with a relative shorter hospital stays [22, 27]. Therefore, the USG-LLI may have an advantage over the UAE in terms of length of hospital stay and adverse event rates.

This meta-analysis showed that USG-LLI and UAE had similar and higher success rates, defined as USG-LLI or UAE combined with curettage, complete recovery without bleeding, fertility preservation, no repeat embolization and surgical intervention, and any serious complications [22, 23]. In addition, hospitalization cost of USG-LLI group was significantly lower than that of the UAE group; this result is conceivable because the UAE is invasive and requires expensive instruments and sophisticated embolic drugs [27].

From the perspective of clinical effect of treatment, the efficacy of USG-LLI in treating CSP is the same as that of UAE. However, USG-LLI was associated with lower complication rates, shorter hospital stays, intervals between menstrual recovery, and lower hospitalization costs. The results of this meta-analysis suggest value for CSP patients who do not want UAE treatment and are seeking alternative therapy.

The Chinese consensus of management on cesarean section pregnancy divides CSP into three types, based on site of implantation, the myometrium thickness between the gestational sac and the posterior wall of the bladder, and the blood flow around gestational sac [39]. The thinner the myometrium between the gestational sac and the richer the blood flow around gestational sac, the higher the risk of the uterine rupture and intraoperative bleeding. Therefore, blind monotherapy is not recommended for high-risk patients. This meta-analysis showed that USG-LLI and UAE had similar and higher success rates, and USG-LLI has more advantages than UAE. But we also need to be aware of its limitations, such as profuse vaginal bleeding or loss of the myometrium between the bladder and sac.

Some of limitations of this meta-analysis will be considered: (1) Most of the included studies are non-randomized controlled, the total number of patients is small and all are Chinese, which means that treatment effect may be overestimated. The caesarean section rate in China is increasing year by year, which provides a strong database support for the epidemiological research of the disease. Additionally, USG-LLI combined with curettage [21] was first reported as a new method for the treatment of CSP in China, and has accumulated a lot of experience. (2) The heterogeneity of some indicators is relatively high, and the random effect model is used for accounting, and the estimation is conservative. (3) Among the studies included in our meta-analysis, follow-up was not very long and did not focus on postoperative fertility outcomes. Therefore, the impact of treatment methods on long-term prognosis and reproductive function of patients remains to be studied.

## Conclusion

USG-LLI was associated with lower complications rates, shorter duration of hospital stays and intervals between menstrual recovery, and lower hospitalization costs. Therefore, the meta-analysis results suggest that USG-LLI may be a more beneficial treatment option than UAE for early treatment of hemodynamically stable CSP. However, the results still need to be further confirmed by large-scale, multi-center randomized controlled trials.

## Data Availability

ALL data can be obtained from the manuscript.

## Abbreviations

CSP: Caesarean scar pregnancy
USG-LLI: Ultrasound-guided local lauromacrogol injection
UAE: Uterine artery embolization
WHO: World Health Organization
MTX: Methotrexate
D&G: Dilatation and curettage
β-hCG: β-Human chorionic gonadotropin
NOS: Newcastle–Ottawa Scale
MD: Mean difference
OR: Odds ratio
Cis: Confidence intervals
